# Transcription factor SP1 regulates haptoglobin fucosylation via induction of GDP-fucose transporter 1 in the hepatoma cell line HepG2

**DOI:** 10.1016/j.bbrep.2022.101372

**Published:** 2022-10-25

**Authors:** Jumpei Kondo, Natsumi Sakata, Koichi Morishita, Ayumu Hayashibara, Daisuke Sakon, Shinji Takamatsu, Nobuhiko Asakura, Takashi Suzuki, Eiji Miyoshi

**Affiliations:** a Department of Molecular Biochemistry and Clinical Investigation, Osaka University Graduate School of Medicine, 1-7 Yamada-oka, Suita, Osaka, 565-0871, Japan; b Center for Mathematical Modeling and Data Science, Osaka University, 1-3 Machikaneyama, Toyonaka, Osaka, 560-8531, Japan

**Keywords:** Fucosylation, Haptoglobin, Solute carrier family 35 member C1(*SLC35C1*), GDP-Fucose transporter 1 (GFT1), Specificity protein 1 (SP1), Cancer biomarker, GDP, Guanosine diphosphate

## Abstract

Fucosylation is involved in cancer and inflammation, and several fucosylated proteins, such as AFP-L3 for hepatocellular carcinoma, are used as cancer biomarkers. We previously reported an increase in serum fucosylated haptoglobin (Fuc-Hp) as a biomarker for several cancers, including pancreatic and colon cancer and hepatocellular carcinoma. The regulation of fucosylated protein production is a complex cellular process involving various fucosylation regulatory genes. In this report, we investigated the molecular mechanisms regulating Fuc-Hp production in cytokine-treated hepatoma cells using a partial least squares (PLS) regression model. We found that *SLC35C1*, which encodes GDP-fucose transporter 1 (GFT1), is the most responsible factor for Fuc-Hp production among various fucosylation regulatory genes. Furthermore, the transcription factor SP1 was essential in regulating *SLC35C1* expression. We also found that an SP1 inhibitor was able to suppress Fuc-Hp production without affecting total Hp levels. Taken together, Fuc-Hp production was regulated by SP1 via induction of GFT1 in the hepatoma cell line HepG2.

## Highlights

• PLS analysis identified *SLC35C1* as a critical gene to promote Hp fucosylation.
• SP1 regulates Fuc-Hp production via inducing *SLC35C1*.
• SP1 inhibition decreases Fuc-Hp production in HepG2 cells.

## 1 Introduction

Fucosylation is one of the most critical glycosylation processes in cancer and inflammation [1], and increased fucosylated proteins in serum can be used as biomarkers of these states. We previously reported that serum from patients with pancreatic cancer shows an increase in fucosylated proteins with a molecular weight of 40 kD, which we identified as haptoglobin (Hp) beta chains [2]. Subsequently, we developed a lectin-antibody enzyme-linked immunosorbent assay (ELISA) for measuring serum fucosylated Hp (Fuc-Hp) and reported that Fuc-Hp is a biomarker for several cancers, including pancreatic, colorectal, and prostate cancer [[3], [4], [5]]. Other groups have also reported that Fuc-Hp is a potential biomarker for non-small cell lung cancer [6,7] and ovarian cancer [8,9]. Recently, we generated a novel glycan antibody that directly recognizes Fuc-Hp and found that Fuc-Hp is produced by hepatocytes surrounding metastatic liver cancer [10]. In chronic liver disease, serum Fuc-Hp levels increase with disease progression [11] and can be a predictive biomarker for hepatocellular carcinoma in patients with chronic hepatitis B and C [12,13]. Fuc-Hp is also the best biomarker for the presence of ballooning hepatocytes in patients with non-alcoholic steatohepatitis [14].

The regulation of protein fucosylation is a complex cellular process involving various fucosylation regulatory genes [1]. The present study investigated the molecular mechanisms regulating Fuc-Hp production in cytokine-treated hepatoma cells using a partial least squares (PLS) regression model. We found that *SLC35C1*, which encodes guanosine diphosphate (GDP)-fucose transporter 1 (GFT), is the most responsible factor among various fucosylation regulatory genes. Furthermore, the transcription factor specificity protein 1 (SP1) was essential in regulating *SLC35C1* expression. We also found that an SP1 inhibitor can suppress Fuc-Hp production without affecting total Hp production.

## 2 Materials and methods

### 2.1 Cell culture

HepG2 (human hepatoma) cells were obtained from American Type Culture Collection (Manassas, VA) and maintained in Dulbecco's modified Eagle medium (DMEM, low-glucose; Nacalai Tesque, Japan) supplemented with 10% fetal bovine serum (FBS; Nichirei Biosciences, Inc., Japan), 100 U/mL penicillin and 100 μg/mL streptomycin (Penicillin-Streptomycin Mixed Solution, Nacalai Tesque) at 37 °C with 5% CO_2_. To stimulate Fuc-Hp production, HepG2 cells cultured in serum-free DMEM (low-glucose) were treated with 0, 0.5, 1, 5, 10, or 20 ng/ml recombinant human interleukin 6 (IL-6; PeproTech, Rocky Hill, NJ). After 6 h, *HP* and fucosylation regulatory gene expression were measured by real-time reverse transcriptase-polymerase chain reaction (RT-PCR), as described below. After 24 h, Fuc-Hp and total Hp protein in the culture supernatant were measured by ELISA, as described below.

Mithramycin A (Abcam, Cambridge, UK) dissolved in dimethyl sulfoxide (DMSO, Wako Pure Chemical Industries, Japan) was diluted with sterilized phosphate-buffered saline (PBS, Nissui Pharmaceutical, Japan) before use. HepG2 cells were cultured in DMEM (low-glucose) with 10% FBS, and various concentrations of mithramycin A (0, 0.1, 0.5, and 1 μM) and 1 ng/ml IL-6 were added. After 8 h, *HP* and fucosylation regulatory gene expression were measured by real-time RT-PCR. After 24 h, both Fuc-Hp and total Hp protein in the culture supernatant were measured by ELISA.

To assess cell viability, HepG2 cells were first cultured with mithramycin A for 24 h, and the CellTiter 96 Aqueous One Solution Cell Proliferation kit (Promega, Madison, WI) was used according to the manufacturer's instructions.

### 2.2 ELISA

We assessed Fuc-Hp concentrations in the culture supernatant using the lectin-antibody ELISA, according to the reported protocol [15]. Briefly, the fragment antigen-binding (Fab) portions of the anti-Hp polyclonal antibody (DAKO, Denmark) were coated onto ELISA plates to capture total Hp. Hp fucosylation was detected by biotinylated *Aleuria aurantia* lectin (AAL) followed by an avidin-peroxidase system. Fuc-Hp levels are described as relative units compared with the Hp levels measured in conditioned medium obtained from PK8 pancreatic cancer cells transfected with an Hp expression vector. To determine the total Hp concentration, the AssayMax™ Human Haptoglobin ELISA kit (Assaypro, St. Charles, MO) was used according to the manufacturer's protocol with control biotinylated human Hp protein diluted 10-fold. Three replicate experiments were performed for each analysis.

### 2.3 Complementary DNA synthesis and real-time RT-PCR

Total RNA was extracted from cells after incubation in TRI REAGENT (Molecular Research Center, Cincinnati, OH). Complementary DNA (cDNA) synthesis was performed with 500 ng total RNA using the Go Script™ Reverse Transcription kit (Promega) and a T100 thermal cycler (BIO-RAD, Hercules, CA). Real-time RT-PCR was performed using THUNDERBIRD™ SYBR® qPCR Mix (TOYOBO, Osaka, Japan) on the Stratagene Mx3000P (Agilent Technologies, Santa Clara, CA) from three replicate experiments. The primers used in this experiment are shown in Supplementary Table 1.

### 2.4 PLS analysis

Partial least squares (PLS regression [16] was used to assess the contributions of *HP* and fucosylation regulatory genes to Hp fucosylation. Standard multiple linear regression can be challenging to perform in the presence of collinearity (high correlation among independent variables) or when using small sample sizes, which was the case for the current data. By contrast, PLS regression can overcome these issues using latent variables (LVs). Specifically, PLS regression extracts a smaller set of LVs that maximize the covariance between independent and dependent variables, leading to a low-rank representation of the data. A PLS regression model was fitted to the current data using the expression of *HP* and fucosylation regulatory genes as independent variables (**X**) and Fuc-Hp concentration as a dependent variable (**y**). PLS regression decomposes the predictor matrix **X** (the columns are the genes, and the rows are the samples) into orthogonal scores **T** and loadings **P** as **X** = **TP** and regresses the response **y** not on **X** itself but on the first *k* columns of **T** (*k* is the number of LVs). In addition, it finds the scores and loadings in such a way to maximize the covariance between **X** and **y**. Leave-one-out cross-validation was conducted to select the optimal number of LVs based on Wold's R criterion [17]. The obtained loadings in each LV were used to identify the gene expression pattern contributing to Hp fucosylation. The influence of each gene in the PLS regression model was assessed using variable importance in projection (VIP) scores [16] and regression coefficients. Genes with a VIP >1 were considered significant for explaining Fuc-Hp concentration variability. The significance of regression coefficients was tested by *t*-test using the Jackknife standard error. PLS analysis was performed using R (version 4.13) with the pls package [18].

### 2.5 Statistical analysis

Statistical analyses were performed using JMP Pro 14 software (SAS Institute Inc., Cary, NC) or R (version 4.13). Multiple comparisons to the control condition were analyzed using Dunnett's test or Student's t-test with Bonferroni correction. A *p*-value less than 0.05 was considered significant.

### 2.6 Database analysis

Candidate transcription factors involved in *SLC35C1* expression were selected from the TFBIND and ChIP-Atlas databases. TFBIND [19] returns possible binding candidates along with DNA sequences. Three *SLC35C1* promoter regions (promoter 1: chr11:45804357–45804416; promoter 2: chr11:45805100–45805159; and promoter 3: chr11:45804034–45804093) obtained from the Eukaryotic Promoter Database [20] were used in this analysis. The ChIP-Atlas [21] database parameters were set as follows: “*H.sapiens*(hg19)”; “ChIP: TFs and others” for Experiment Type; “Liver” for Cell Type Class; “100” for Threshold for Significance; “SLC35C1” for Enter Dataset; “−5000 bp ≤ transcription start site (TSS) ≤ +5000 bp” for distance range from TSS. *P*-values were calculated with the two-tailed Fisher's exact probability test.

## 3 Results

### 3.1 PLS analysis identifies *SLC35C1* as a critical gene to promote Hp fucosylation

**Fig. 1:**
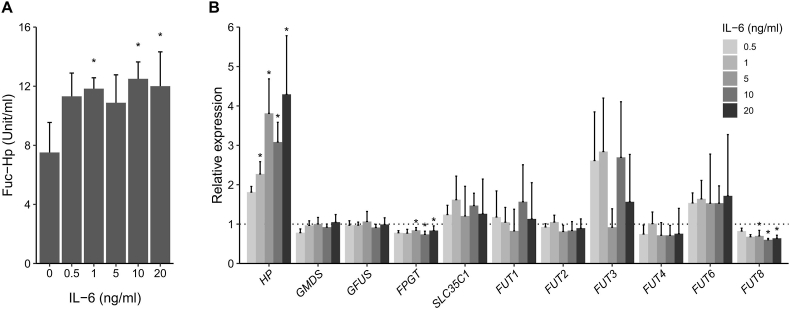
**Changes in Fuc-Hp and candidate regulatory gene expression upon IL-6 stimulation.** A) Fuc-Hp levels in the culture media of IL-6–stimulated HepG2 cells were quantified by AAL ELISA. Data are presented as the average ± S.D. (N = 3 for each condition). *p < 0.05. B) Relative expression of *HP* and fucosylation regulatory genes in HepG2 cells stimulated by IL-6. Data are normalized to unstimulated (0 ng/ml IL-6) cells and presented as the average ± S.D. (N = 3 for each condition). *p < 0.05.

AAL ELISA revealed that IL-6 treatment significantly increased Fuc-Hp production in a dose-dependent manner (Fig. 1A). RT-PCR revealed a marked enhancement in *HP* expression and a slight reduction in *FPGT* and *FUT8* expression after IL-6 treatment in a dose-dependent manner (Fig. 1B).

**Fig. 2:**
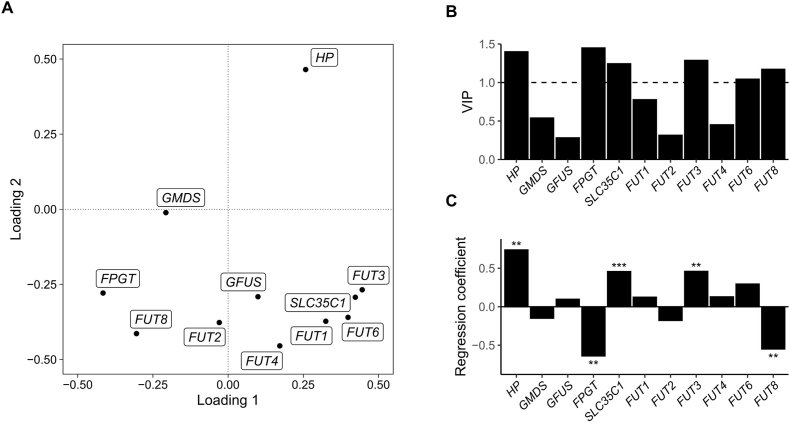
PLS analysis results. A) Plot of the loadings for *HP* and fucosylation regulatory gene expression for the two latent variables (loadings 1 and 2). B) Variable importance in projection scores. C) Regression coefficients. *p < 0.05, **p < 0.01, ***p < 0.001.

To evaluate the relative contributions of *HP* and fucosylation regulatory genes to Fuc-Hp production, we used PLS regression to construct an LV model that parsimoniously explains the covariance between the response variable (Fuc-Hp) and the independent variables (*HP* and fucosylation regulatory gene expression). PLS analysis resulted in a model consisting of two significant LVs that explained 65% of the variance in the independent variables. Fig. 2A shows the loadings of the LVs for *HP* and fucosylation regulatory gene expression, in which positive and negative loadings indicate positive and negative correlations with Fuc-Hp production, respectively [22]. For the first LV, positive loadings include *GFUS* and *SLC35C1*. Moriwaki et al. [23] reported that high expression levels of these genes induced high fucosylation levels in HepG2 cells. Thus, the first LV can be interpreted as representing fucosylation due to IL-6 treatment. *HP* expression was the only variable with a positive and large loading for the second LV, suggesting that the second LV represents the effect of Hp production. The second loadings were negative for all other fucosylation regulatory genes, suggesting that *HP* upregulation is associated with the downregulation of other fucosylation regulatory genes. Our PLS analysis identified two separate processes for fucosylation and Hp production that contribute to Hp fucosylation.

We then quantified the importance of *HP* and fucosylation regulatory gene expression for overall Fuc-Hp production using VIP scores and regression coefficients. Fig. 2B shows the VIP scores for *HP* and fucosylation regulatory gene expression. The significant genes (VIP >1) are *HP*, *FPGT*, *SLC35C1*, *FUT3*, *FUT6*, and *FUT8*, and the regression coefficients (Fig. 2C) indicate that *SLC35C1* is the most significant and positive predictor of Fuc-Hp production. Therefore, we conclude that *SLC35C1* is a critical gene responsible for IL-6–stimulated Fuc-Hp production in HepG2 cells.

### 3.2 Database analysis suggests SP1 as a transcription factor that regulates Fuc-Hp production

**Fig. 3:**
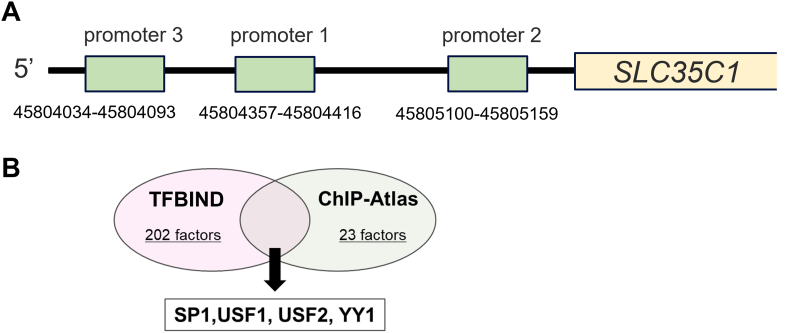
Database analysis extracted SP1 as a candidate regulatory protein for *SLC35C1*. A) A schema of the *SLC35C1* promoter region obtained from the database. B) A Venn diagram of the possible binding transcription factors extracted from the promoter sequence of *SLC35C1* obtained using TFBIND and ChIP-Atlas.

As PLS analysis showed a positive correlation between *SLC35C1* expression and Hp fucosylation, we next sought to identify upstream transcription factors that regulate *SLC35C1* expression using two databases: TFBIND and ChIP-Atlas. We obtained three *SLC35C1* promoter region sequences from the Eukaryotic Promoter Database (Fig. 3A) and analyzed these sequences with both TFBIND and ChIP-Atlas to identify potential transcription factors that bind with each sequence (Fig. 3B). We identified four transcription factors in both databases, including SP1, which was previously reported to regulate *SLC35C1* expression in HEK293 cells [24]. Therefore, we further examined the regulation of Hp fucosylation by SP1 in HepG2 cells.

### 3.3 SP1 inhibition decreases the production of Fuc-Hp in HepG2 cells

**Fig. 4:**
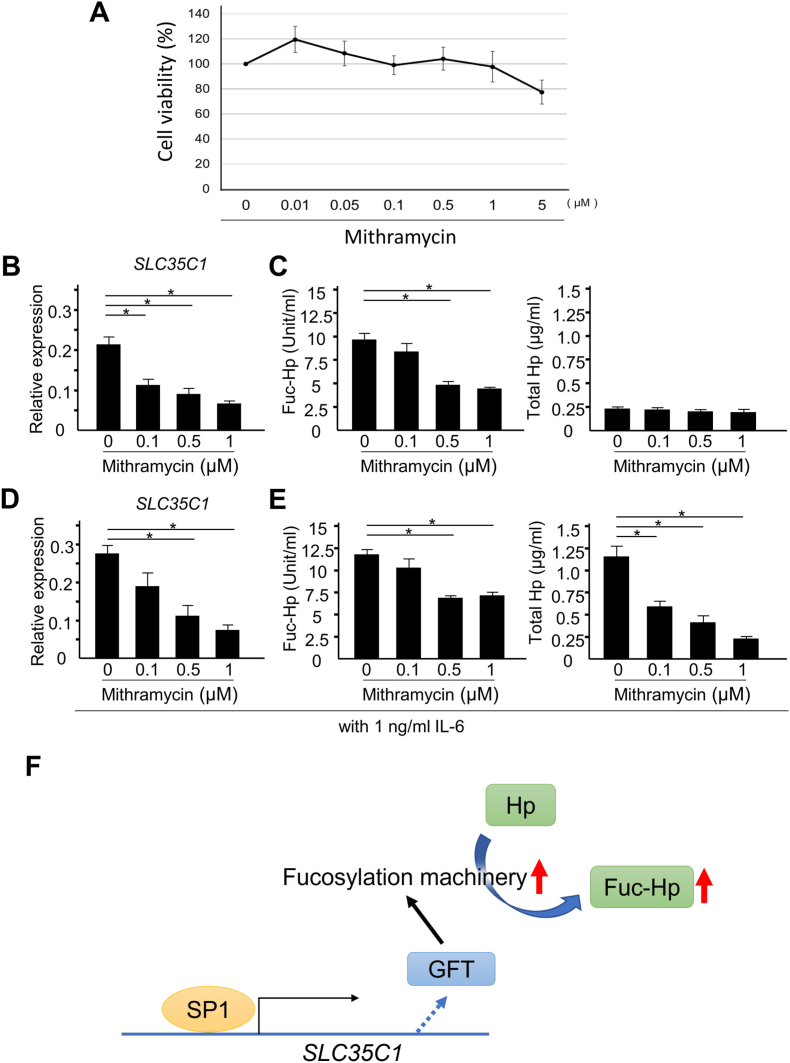
Mithramycin A suppresses Fuc-Hp production at the fucosylation level in HepG2 cells. A) Viability of HepG2 cells treated with increasing doses of mithramycin A. Cell viability was normalized to vehicle control. Data are shown as the average ± S.D. (N = 3 for each condition). B) *SLC35C1* expression in HepG2 cells treated with mithramycin A. *RPL4* was used as the reference gene, and data are shown as the average ± S.D. (N = 3 for each condition). *p < 0.05. C) The concentration of Fuc-Hp (left) and total Hp (right) in the culture supernatant of HepG2 cells treated with mithramycin A. Data are presented as the average ± S.D. (N = 3 for each condition). *p < 0.05. D) *SLC35C1* expression in HepG2 cells stimulated with IL-6 and treated with mithramycin A. *RPL4* was used as the reference gene, and data are shown as the average ± S.D. (N = 3 for each condition). E) The concentration of Fuc-Hp (left) and total Hp (right) in the culture supernatant of HepG2 cells stimulated by IL-6 and treated with mithramycin A. Data are presented as the average ± S.D. (N = 3 for each condition). *p < 0.05. F) A schematic illustration describing the SP1-mediated *SLC35C1*/GFT induction activates the protein fucosylation machinery to upregulate the Fuc-Hp level.

To examine the contribution of SP1 to Hp fucosylation, Fuc-Hp production was evaluated in HepG2 cells treated with the SP1 inhibitor mithramycin A, which interrupts SP1 binding to DNA. Because mithramycin A is reported to be cytotoxic in certain cells, we evaluated the effect of mithramycin A on HepG2 cell viability [25]. Mithramycin A at concentrations as high as 1 μM had no growth inhibitory effects in HepG2 cells (Fig. 4A). As expected from the database analysis, *SLC35C1* expression was significantly suppressed by mithramycin A in a dose-dependent manner (Fig. 4B), along with the suppression of known SP1 target gene *VEGFA* [26] (Fig. S1A). Consistent with this finding, the production of Fuc-Hp was also suppressed by 0.5 and 1 μM mithramycin A (Fig. 4C left). Of note, mithramycin A also suppressed the expression of the other fucosylation-related genes, such as *FUT6* and *FUT8* (Fig. S1B), supporting the potential of SP1 inhibition to suppress fucosylation levels in HepG2 cells. Importantly, mithramycin A showed no suppressive effect on the concentration of total Hp (Fig. 4C right), suggesting that SP1 inhibition suppressed Fuc-Hp production at the fucosylation level in HepG2 cells.

We next evaluated the effect of mithramycin A under a cytokine-stimulated condition because our mathematical analysis was based on the IL-6–stimulated production of Fuc-Hp. In HepG2 cells stimulated with 1 ng/ml of IL-6, mithramycin A significantly decreased *SLC35C1* expression in a dose-dependent manner (Fig. 4D). Accordingly, the IL-6–stimulated production of Fuc-Hp in HepG2 cells was suppressed by 0.5 and 1 μM mithramycin A (Fig. 4E left). Under IL-6–stimulated conditions, total Hp production was also suppressed by 0.1, 0.5 and 1 μM mithramycin A (Fig. 4E right).

## 4 Discussion

We previously reported higher fucosylation levels in HepG2 cells than in other hepatoma cell lines due to the increased expression of *SLC35C1* mRNA, which encodes GFT [23]. In the present study, we showed that SP1-mediated *SLC35C1*/GFT expression regulates Fuc-Hp production in HepG2 cells (Fig. 4F). Additionally, we used PLS regression to demonstrate that *SLC35C1* is a crucial regulator of Hp fucosylation in IL-6–stimulated HepG2 cells and identified the fucosyltransferases *FUT3*, *FUT6*, and *FUT8* as significant contributors to the PLS model. The positive regression coefficients for *FUT3* and *FUT6* (Fig. 2C) suggest that the IL-6–mediated increase in Fuc-Hp is due to alpha-1,3/1,4–linked fucosylation. By contrast, the negative coefficient for *FUT8* implies a reduction in alpha-1,6 fucosylation (core fucosylation) after IL-6 stimulation, which does not contradict our previous finding [27] that *FUT8* overexpression does not facilitate cellular fucosylation in hepatoma cells. We hypothesize that under IL-6–stimulated conditions, the suppression of *FUT8* expression plays a homeostatic role in regulating Fuc-Hp production, which may explain why Fuc-Hp showed a saturated increase (Fig. 1A), despite an almost linear increase in its donor substrate following IL-6 treatment (Fig. 1B).

We also found that SP1 is the primary transcription factor responsible for *SLC35C1* expression in HepG2 cells. As shown in Fig. 4, in the absence of cytokine stimulation, the SP1 inhibitor mithramycin A inhibited *SLC35C1* expression and suppressed Fuc-Hp production in HepG2 cells without affecting *HP* expression, possibly through the downregulation of GFT. In addition, some of the other fucosylation-related genes, such as *FUT6* and *FUT8*, were also suppressed with mithramycin A treatment. These results that mithramycin targets multiple fucosylation-related genes in HepG2 cells support the potential of SP1 inhibition as a strategy to suppress fucosylation levels in hepatocellular carcinoma. IL-6 upregulates Fuc-Hp production via the induction of both *HP* and fucosylation regulatory gene expression [28], and the effects of IL-6 can also be suppressed by SP1 inhibition (Fig. 4), associated with decreases in both *HP* and *SLC35C1* expression. Previous studies have shown that *HP* expression can be upregulated by IL-6 signaling via the signal transducer and activator transcription 3 (STAT3) pathway [29], which can crosstalk or synergize with SP1-mediated transcription [[30], [31], [32]]. Thus, SP1 may be responsible for IL-6–stimulated *HP* upregulation but not the basal expression of *HP* in HepG2 cells in the absence of cytokine stimulation.

Whether SP1-mediated GFT production is responsible for Hp fucosylation in the various clinical conditions for which Fuc-Hp can be used as a biomarker remains unclear. Whether high Fuc-Hp levels in cancer are only a marker for disease progression or whether Fuc-Hp is involved in disease progression is also unclear. If Fuc-Hp plays a role in promoting cancer progression, the inhibition of Fuc-Hp production or function may have therapeutic potential, and SP1 inhibition may represent a potential treatment option.

## Funding

This work was supported by JSPS KAKENHI [grant numbers 19H03562, 22H02967] and 10.13039/100009619AMED [grant number 21fk0210079h0002].

## Declaration of competing interest

The authors declare that they have no known competing financial interests or personal relationships that could have appeared to influence the work reported in this paper.

## Data Availability

Data will be made available on request.
