# Supplementary material for: Transcription factor SP1 regulates haptoglobin fucosylation via induction of GDP-fucose transporter 1 in the hepatoma cell line HepG2
Source: Biochem Biophys Rep. 2022 Oct 25;32:101372. doi: 10.1016/j.bbrep.2022.101372 (PMC9615130; doi:10.1016/j.bbrep.2022.101372)
Supplement: Multimedia component 2 [file mmc2.pdf]

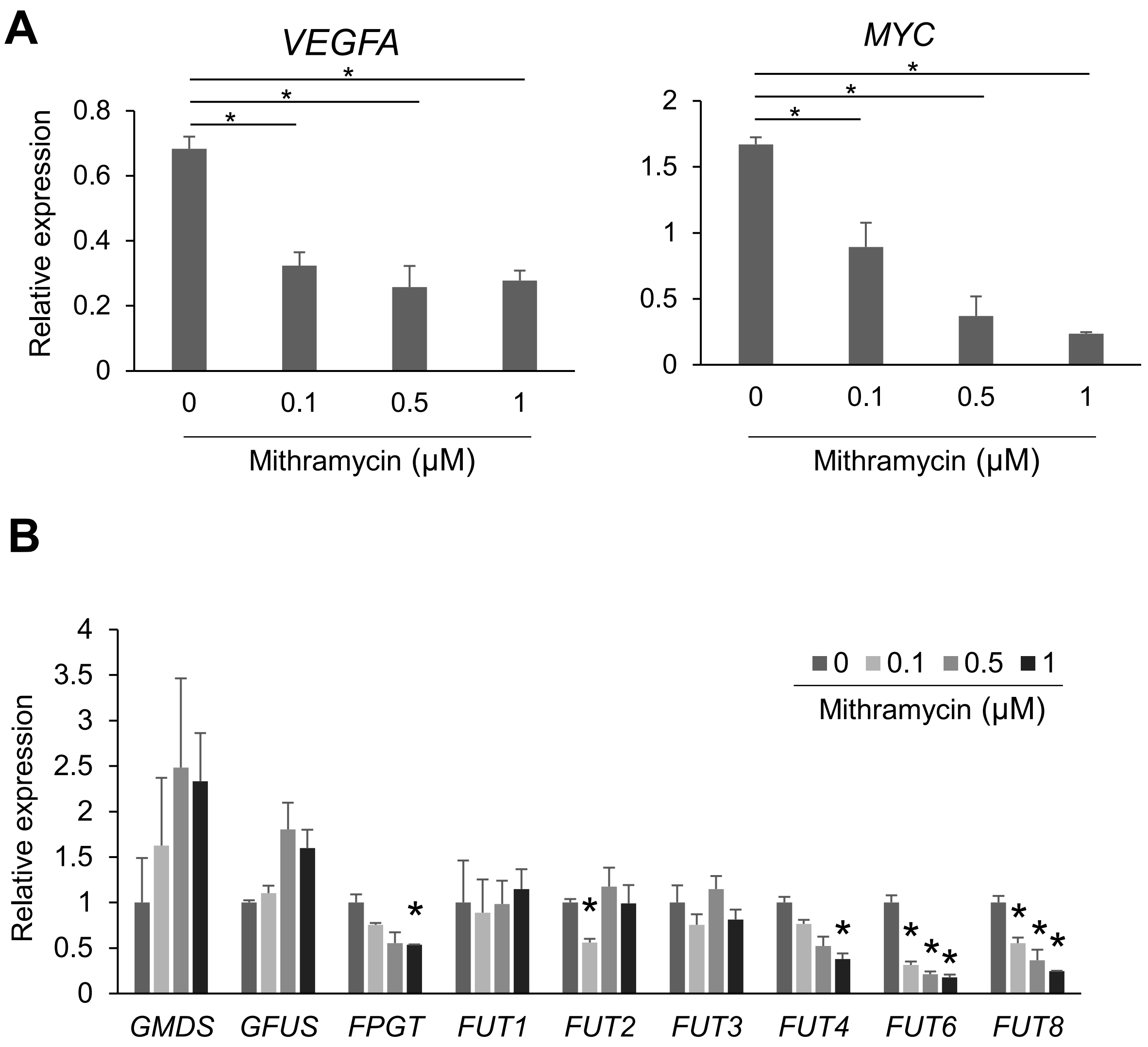

Figure S1

Expression of *VEGFA, MYC* (A), and fucosylation-related genes (B) in HepG2 cells treated with mithramycin A. *RPL4* was used as the reference gene, and data are shown as the average ± S.D. (N = 3 for each condition). Student's t-test with Bonferroni correction, * $p < 0.05$, comparing with 0μM mithramycin in each gene.
